# Author Correction: Specific heterozygous variants in *MGP* lead to endoplasmic reticulum stress and cause spondyloepiphyseal dysplasia

**DOI:** 10.1038/s41467-024-47898-x

**Published:** 2024-04-30

**Authors:** Ophélie Gourgas, Gabrielle Lemire, Alison J. Eaton, Sultanah Alshahrani, Angela L. Duker, Jingjing Li, Ricki S. Carroll, Stuart Mackenzie, Sarah M. Nikkel, Care4Rare Canada Consortium, Michael B. Bober, Kym M. Boycott, Monzur Murshed

**Affiliations:** 1 Department of Medicine, McGill University, Montreal, QC Canada; 2 Children’s Hospital of Eastern Ontario Research Institute, University of Ottawa, Ottawa, ON Canada; 3 Department of Genetics, Children’s Hospital of Eastern Ontario, Ottawa, ON Canada; 4 Broad Center for Mendelian Genomics, Program in Medical and Population Genetics, Broad Institute of MIT and Harvard, Cambridge, MA USA; 5 University of Alberta, Edmonton, AB Canada; 6 Faculty of Dental Medicine and Oral Health Sciences, McGill University, Montreal, QC Canada; 7 Department of Oral and Maxillofacial Surgery, Faculty of Dentistry, King Abdulaziz University, Jeddah, Saudi Arabia; 8 Nemours Children’s Health, Wilmington, DE USA; 9 University of British Columbia, Vancouver, BC Canada; 10 Shriners Hospitals for Children ˗ Canada, Montreal, QC Canada

**Keywords:** Diseases, Gene expression, Bone development, Cartilage development, CRISPR-Cas9 genome editing

Correction to: *Nature Communications* 10.1038/s41467-023-41651-6, published online 3 November 2023

In this article, the author name Alison J. Eaton was incorrectly written as Alison L. Eaton. The original article has been corrected.

