# Supplementary material for: Specific heterozygous variants in MGP lead to endoplasmic reticulum stress and cause spondyloepiphyseal dysplasia
Source: Nat Commun. 2023 Nov 3;14:7054. doi: 10.1038/s41467-023-41651-6 (PMC10624854; doi:10.1038/s41467-023-41651-6)
Supplement: Supplementary file 1 — Supplementary Information [file 41467_2023_41651_MOESM1_ESM.pdf]

Supplementary Figures

Supplementary Figure 1

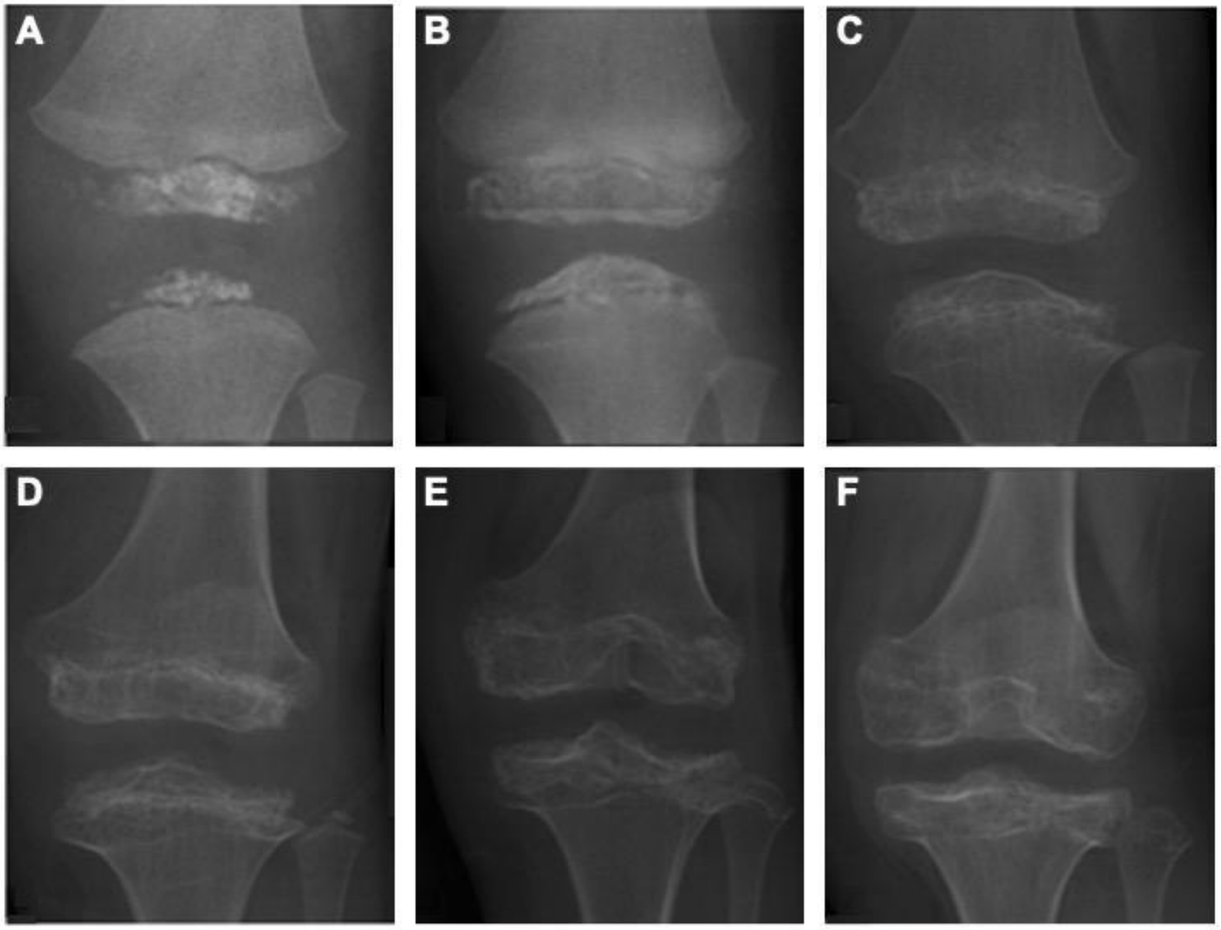

Supplementary Figure 1. **The evolution of physeal and epiphyseal changes in SED, MGP Type.** Standing Anterior-Posterior (AP) Radiographs of the left knee on Individual 4 taken at ages: (A) 2 years 4 months; (B) 3 years 9 months; (C) 5 years 10 months; (D) 7 years 2 months; (E) 10 years 5 months; and (F) 11 years 0 months. The epiphyses began as small for age and with fragmentation, they gradually mineralized and enlarged with areas of patchy sclerosis. The final epiphyseal thickness was diminished. Between the ages of 7 and 10 years, the physes of the distal femur and proximal radius began to fuse with no visible remaining growth plate seen at 11 years of age.

Supplementary Figure 2

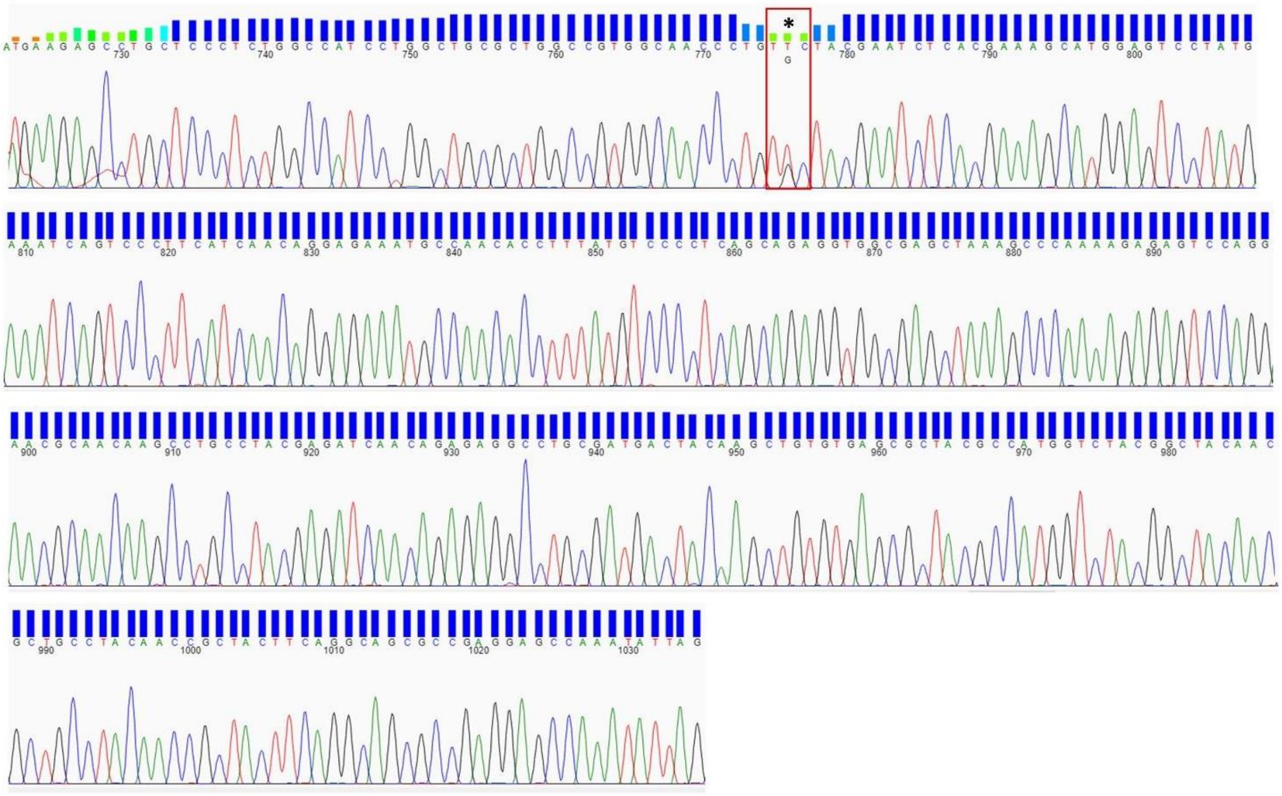

Supplementary Figure 2. Sanger sequencing analysis of the cDNAs synthesized from the total RNA extracted from the heart of a *Mgp*$^{+/56G>T}$ mouse. No undesirable 'off-target' mutations have been introduced in the *Mgp* gene during our DNA manipulation method. Note the double peaks within the red box representing the guanine and thymine nucleotides at position 56. A reverse primer P3 (5'- AAGTGAACGTATCAAACCAGAAT-3') was used for sequencing and the sequence was analyzed using the Nanuq web application available via Genome Quebec.

Supplementary Figure 3

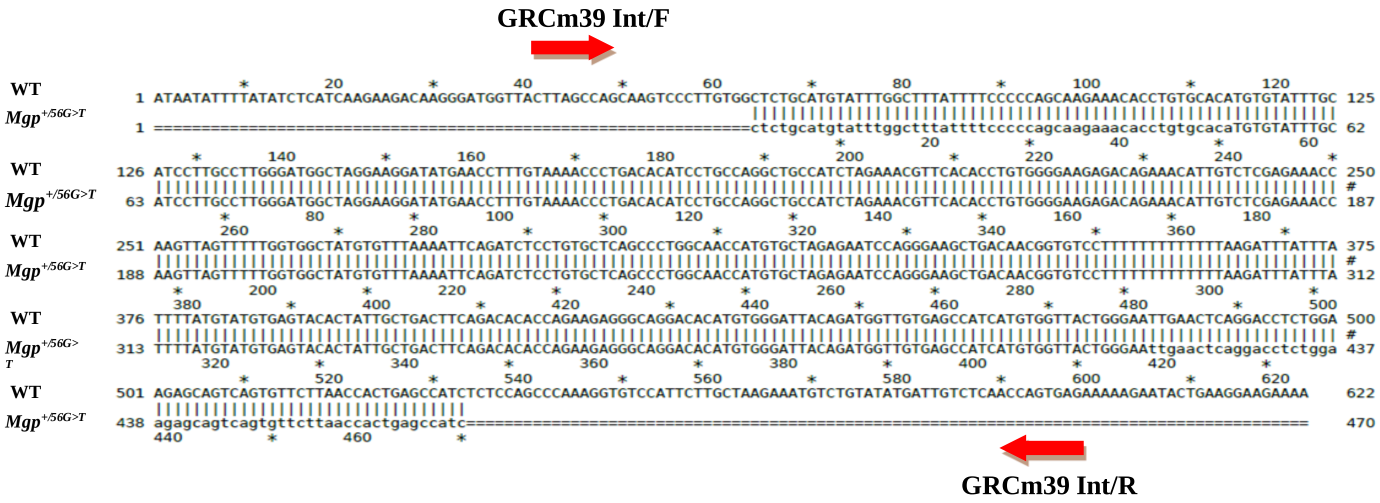

Supplementary Figure 3. No 'off-target effect' in generated *Mgp*$^{+/56G>T}$ mouse genome. One region (GRCm39) in the mouse genome was identified by CRISPOR application for "off-target" prediction for guide RNAs. We designed specific primer pairs (GRCm39 Int/F and GRCm39 Int/R) to amplify the possible off-target sequence and performed Sanger sequencing of the purified amplicons. As shown in the above sequence comparison, no alterations of the amplicon sequence were detected when compared with the wild type sequence. DNA Strider software was used for sequence comparison.

Supplementary Figure 4

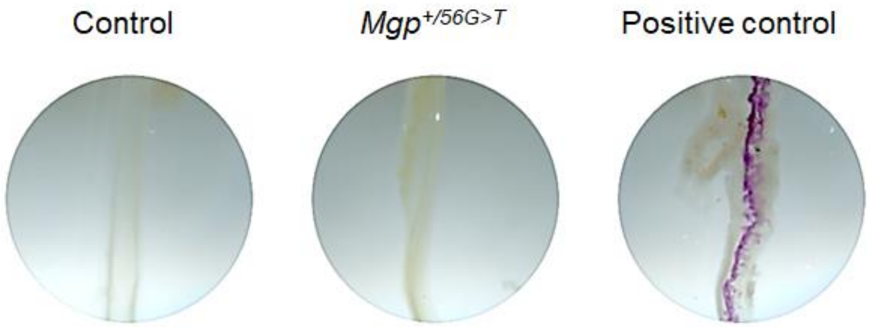

Supplementary Figure 4. Alizarin red staining of the thoracic aorta from control and *Mgp*$^{+/56G>T}$ mice. Note that no sign of thoracic aorta calcification is present in *Mgp*$^{+/56G>T}$ mice. Thoracic aorta from a mouse lacking functional MGP was used as a positive control. All mice were 6-week-old at the time of sample collection. Aorta samples were fixed overnight in 95% ethanol, treated with 2% potassium hydroxide for 24 hours and then stained by 0.005% Alizarin Red (Sigma-Aldrich) in a 1% potassium hydroxide solution. Finally, the stained aorta tissues were clarified in 1% potassium hydroxide/20% glycerol for 2 days.

Supplementary Figure 5

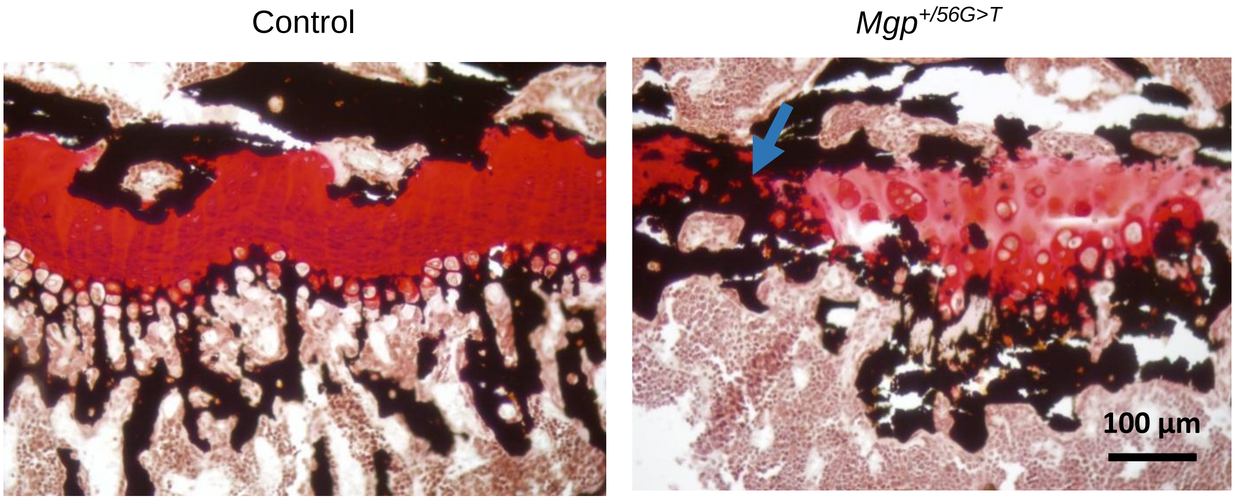

Supplementary Figure 5. C19F variant in MGP results in growth plate abnormalities in the long bones. Von Kossa and safranin O (VKSO) staining of the tibia sections from 6-week-old control and *Mgp*$^{+/56G>T}$ mice. The growth plates of *Mgp*$^{+/56G>T}$ mice are abnormally mineralized (arrow) with disorganized cellular distribution and weaker safranin O staining of the extracellular matrix.

Supplementary Figure 6

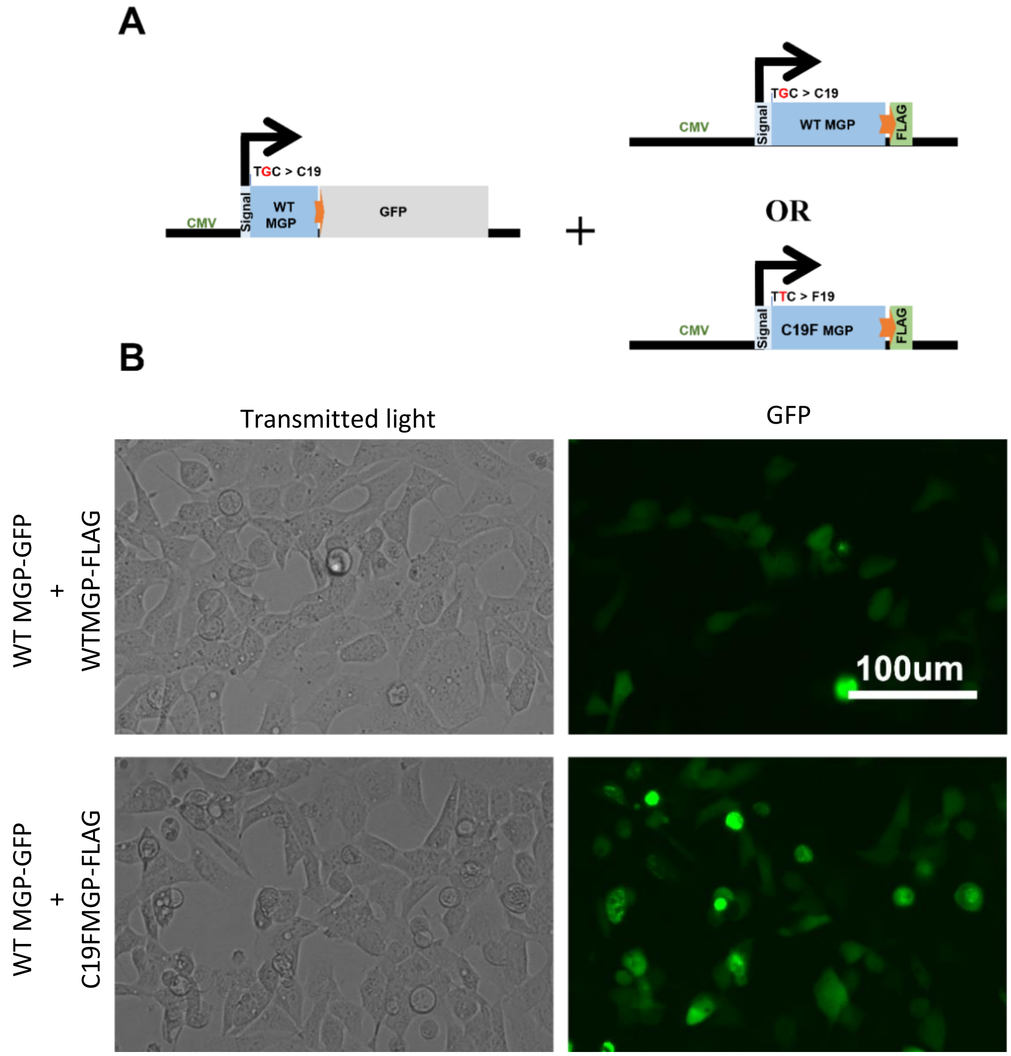

Supplementary Figure 6. C19F MGP protein affects the secretion of wild type protein. A. Scheme for the co-expression of wild type MGP-GFP fusion protein together with WT MGP-FLAG or C19F-MGP-FLAG protein in HEK-293 cells. B. When co-expressed with C19F-MGP-FLAG protein, MGP-GFP protein shows stronger cellular signal than when co-expressed with WT MGP-FLAG protein. This observation suggests that the mutant protein promotes the retention of the MGP-GFP protein.

Care4Rare Canada Consortium

Kym Boycott (lead; University of Ottawa), Michael Brudno (co-lead,University of Toronto), Francois Bernier (co-lead, University of Calgary), Clara van Karnebeek (co-lead,University of British Columbia), David Dyment (CHEO Research Institute), Kristin Kernohan (Newborn Screening Ontario, University of Ottawa), Micheil Innes (University of Calgary), Ryan Lamont (University of Calgary), Jillian Parboosingh (University of Calgary), Deborah Marshall (University of Calgary), Christian Marshall (University of Toronto), Roberto Mendoza (University of Toronto), James Dowling (University of Toronto), Robin Hayeems (University of Toronto), Bartha Knoppers (McGill University), Anna Lehman (University of British Columbia), Sara Mostafavi (University of British Columbia).
