# Supplementary material for: Specific heterozygous variants in MGP lead to endoplasmic reticulum stress and cause spondyloepiphyseal dysplasia
Source: Nat Commun. 2023 Nov 3;14:7054. doi: 10.1038/s41467-023-41651-6 (PMC10624854; doi:10.1038/s41467-023-41651-6)
Supplement: Supplementary file 3 — Description of Additional Supplementary Files [file 41467_2023_41651_MOESM3_ESM.pdf]

Supplementary Data 1: Additional information on the four individuals affected by spondyloepiphyseal dysplasia, MGP type and their detailed phenotypic features.
