# Supplementary material for: Specific heterozygous variants in MGP lead to endoplasmic reticulum stress and cause spondyloepiphyseal dysplasia
Source: Nat Commun. 2023 Nov 3;14:7054. doi: 10.1038/s41467-023-41651-6 (PMC10624854; doi:10.1038/s41467-023-41651-6)
Supplement: Supplementary file 5 — Reporting Summary [file 41467_2023_41651_MOESM5_ESM.pdf]

nature portfolio

Corresponding author(s): Monzur Murshed, PhD

Last updated by author(s): Jun 1, 2023

Reporting Summary

Nature Portfolio wishes to improve the reproducibility of the work that we publish. This form provides structure for consistency and transparency in reporting. For further information on Nature Portfolio policies, see our Editorial Policies and the Editorial Policy Checklist.

Statistics

For all statistical analyses, confirm that the following items are present in the figure legend, table legend, main text, or Methods section.

n/a	Confirmed	
☐	☒	The exact sample size (*n*) for each experimental group/condition, given as a discrete number and unit of measurement
☐	☒	A statement on whether measurements were taken from distinct samples or whether the same sample was measured repeatedly
☐	☒	The statistical test(s) used AND whether they are one- or two-sided<br>*Only common tests should be described solely by name; describe more complex techniques in the Methods section.*
☒	☐	A description of all covariates tested
☒	☐	A description of any assumptions or corrections, such as tests of normality and adjustment for multiple comparisons
☐	☒	A full description of the statistical parameters including central tendency (e.g. means) or other basic estimates (e.g. regression coefficient) AND variation (e.g. standard deviation) or associated estimates of uncertainty (e.g. confidence intervals)
☐	☒	For null hypothesis testing, the test statistic (e.g. *F*, *t*, *r*) with confidence intervals, effect sizes, degrees of freedom and *P* value noted<br>*Give P values as exact values whenever suitable.*
☒	☐	For Bayesian analysis, information on the choice of priors and Markov chain Monte Carlo settings
☒	☐	For hierarchical and complex designs, identification of the appropriate level for tests and full reporting of outcomes
☒	☐	Estimates of effect sizes (e.g. Cohen's *d*, Pearson's *r*), indicating how they were calculated

Our web collection on statistics for biologists contains articles on many of the points above.

Software and code

Policy information about availability of computer code

Data collection	NRecon, 3CTAn, and CTVol (SkyScan; version 1.20.8.0), DP2-BSW software (Olympus; version 2.2), Photoshop (Adobe, version 12, 0.04 x32).
Data analysis	DNA Strider (version 3.5 z1), SnapGene Viewer, (version 6.0.2) Scaffold Q+ Scafflold (Proteome Sciences, version 4.4.8), Pinnacle (Optys Tech), ImageJ (National Institutes of Health, version 1.53t)) GraphPad Prism (version 7.04), Osteomeasure7 (OsteoMetrics, version 4.3.0.0).

For manuscripts utilizing custom algorithms or software that are central to the research but not yet described in published literature, software must be made available to editors and reviewers. We strongly encourage code deposition in a community repository (e.g. GitHub). See the Nature Portfolio guidelines for submitting code & software for further information.

Data

Policy information about availability of data

All manuscripts must include a data availability statement. This statement should provide the following information, where applicable:

- Accession codes, unique identifiers, or web links for publicly available datasets
- A description of any restrictions on data availability
- For clinical datasets or third party data, please ensure that the statement adheres to our policy

All data generated for this study are available within the manuscript and its supplementary Information. Also source data files contain the relevant raw data. Proteomic data have been uploaded to public repository.

Research involving human participants, their data, or biological material

Policy information about studies with human participants or human data. See also policy information about sex, gender (identity/presentation), and sexual orientation and race, ethnicity and racism.

Reporting on sex and gender	Biological sex of the patients carrying the mutations was indicated.
Reporting on race, ethnicity, or other socially relevant groupings	Self-reported ancestry was used in the analysis of the patient data.
Population characteristics	Four individuals affected with spondyloepiphyseal dysplasia were studied in this project (3 females, 10, 18 and 52 years old; 1 male 12 yrs old). Please see the supplementary table 1.
Recruitment	The three individuals from Family 1 were enrolled in a research consortium on the genomics of rare disease, Care4Rare Canada. The fourth individual from Family 2 was recruited in this study by his clinical team. Informed consent to participate was obtained from all individuals. The individuals were recruited as they visited the hospital for the treatment.
Ethics oversight	This study was approved by the Children's Hospital of Eastern Ontario Research Ethics Board. Consent to publish was obtained for all individuals.

Note that full information on the approval of the study protocol must also be provided in the manuscript.

Field-specific reporting

Please select the one below that is the best fit for your research. If you are not sure, read the appropriate sections before making your selection.

☒ Life sciences ☐ Behavioural & social sciences ☐ Ecological, evolutionary & environmental sciences

For a reference copy of the document with all sections, see nature.com/documents/nr-reporting-summary-flat.pdf

Life sciences study design

All studies must disclose on these points even when the disclosure is negative.

Sample size	For in vitro and in vivo experiments n=3 to 6 or more samples were used. In most cases, experiments were repeated at least twice with 3 or more replicates each time. Suitability of the sample sizes was determined by power calculations using a web resource for statistical analyses (http://www.openepi.com).
Data exclusions	No data were exculded.
Replication	For each experiments at least three replicates were used. All attempts at replication generated reproducible results supporting the overall conclusion.
Randomization	For the in vivo experiments, we determined the genotypes for the control and experimental groups and selected them randomly for the experiments described in the manuscript. We performed comparative analyses on control and mutant mice keeping all the variables identical (except the genotype). For cell culture experiments same batch of cells were plated and the wells were grouped randomly for each experimental parameters.
Blinding	N/A; for each experiments, the differences in compared parameters were readily noticeable. Moreover collected data were independently verified by more than 2 co-authors.

Reporting for specific materials, systems and methods

We require information from authors about some types of materials, experimental systems and methods used in many studies. Here, indicate whether each material, system or method listed is relevant to your study. If you are not sure if a list item applies to your research, read the appropriate section before selecting a response.

Materials & experimental systems

n/a	Involved in the study
	☒ Antibodies
	☒ Eukaryotic cell lines
☒	Palaeontology and archaeology
	☒ Animals and other organisms
☒	Clinical data
☒	Dual use research of concern
☒	Plants

Methods

n/a	Involved in the study
☒	ChIP-seq
☒	Flow cytometry
☒	MRI-based neuroimaging

Antibodies

Antibodies used

1) DYKDDDDK Tag (D6W5B) Rabbit mAb (Binds to same epitope as Sigma's Anti-FLAG® M2 Antibody, Cell Signaling Technology, Catalog number#14793, Lot number#7.
2) ANTI-FLAG® M2 Monoclonal mouse antibody, Millopore Sigma, Catalog number#F1804-200UG, Lot number# SLCD6338.
3) Anti-Collagen II antibody, abcam, Catalog number# ab21291, Lot number# GR50036-2.
4) Anti-Aggrecan antibody, abcam, Catalog number# ab36861, Lot number# GR104534-1.
5) Recombinant Anti-Collagen X antibody [EPR22920-211], abcam, Catalog number# ab260040, Lot number# GR3387885-6.
6) Calnexin (C5C9) Rabbit mAb, Cell Signaling Technology, Catalog number #2679, Lot number# N/A.
7) CHOP (L63F7) Mouse mAb, Cell Signaling Technology, Catalog number #2895, Lot number#13.
8) XBP-1s (D2C1F) Rabbit mAb, Cell Signaling Technology, Catalog number #12782, Lot number#6.
9) Phospho-eIF2α (Ser51) (D9G8) XP® Rabbit mAb, Cell Signaling Technology, Catalog number #3398, Lot number#6.
10) Anti-ATF6 antibody, abcam, Catalog number# ab37149, Lot number# N/A.
11) Cy™3 AffiniPure Donkey Anti-Rabbit IgG (H+L), Jackson ImmunoResearch, Catalog number 711-165-152, Lot number# 117211.
12) DyLight 488-conjugated Donkey Anti Rabbit IgG, Jackson ImmunoResearch, Catalog number 711-486-152, Lot number# 80741.
13) Alexan Fluor488-conjugated Goat Anti-mouse IgG Fab2, Cell Signaling Technology, Catalog number #4408S, Lot number# N/A.
14) Alexan Fluor594-conjugated Goat Anti-mouse IgG Fab2, Cell Signaling Technology, Catalog number #8890S, Lot number# N/A.
15) Anti-rabbit IgG, HRP-linked Antibody, Cell Signaling Technology, Catalog number #7074S, Lot number# N/A.

Validation

We chose the antibodies based on the validated data provided by the manufacturers and/or the published literature. Please see below the links describing the product data sheet and publications reporting the antibodies.
1) DYKDDDDK Tag (D6W5B) Rabbit mAb detects exogenously expressed DYKDDDDK proteins in cells. The antibody recognizes the DYKDDDDK peptide, which is the same epitope recognized by Sigma's Anti-FLAG® antibodies, fused to either the amino-terminus or carboxy-terminus of the target protein. Anti-FLAG® M2 Antibody has been validated using SimpleChIP® Enzymatic Chromatin IP Kits (https://www.cellsignal.com/products/primary-antibodies/dykddddk-tag-d6w5b-rabbit-mab-binds-to-same-epitope-as-sigma-s-anti-flag-m2-antibody/14793).
2) ANTI-FLAG® M2 mouse affinity purified monoclonal antibody binds to fusion proteins containing a FLAG peptide sequence. The antibody recognizes the FLAG peptide sequence at the N-terminus, Met-N-terminus,C-terminus, and internal sites of the fusion protein. https://www.sigmaaldrich.com/deepweb/assets/sigmaaldrich/product/documents/175/747/f1804bul-ms.pdf.
3) Anti-Collagen II antibody, PubMed: 21177286 and https://www.abcam.com/products/primary-antibodies/collagen-ii-antibody-ab21291.html
4) Anti-Aggrecan antibody, https://www.abcam.com/products/primary-antibodies/aggrecan-antibody-ab36861.html
5) Recombinant Anti-Collagen X antibody [EPR22920-211], https://www.abcam.com/products/primary-antibodies/collagen-x-antibody-epr22920-211-ab260040.html
6) Calnexin (C5C9) Rabbit mAb #2679, https://www.cellsignal.com/products/primary-antibodies/calnexin-c5c9-rabbit-mab/2679?_=1685476197960&Ntt=2679&tahead=true.
7) CHOP (L63F7) Mouse mAb #2895, https://www.cellsignal.com/products/primary-antibodies/chop-l63f7-mouse-mab/2895?_=1685478763078&Ntt=2895&tahead=true.
8) XBP-1s (D2C1F) Rabbit mAb #12782, https://www.cellsignal.com/products/primary-antibodies/xbp-1s-d2c1f-rabbit-mab/12782.
9) Phospho-eIF2α (Ser51) (D9G8) XP® Rabbit mAb #3398, https://www.cellsignal.com/products/primary-antibodies/phospho-eif2a-ser51-d9g8-xp-rabbit-mab/3398.
10) Anti-ATF6 antibody (ab37149), https://www.abcam.com/products/primary-antibodies/atf6-antibody-ab37149.html
11) Cy™3 AffiniPure Donkey Anti-Rabbit IgG (H+L) (Jackson ImmunoResearch, 711-165-152) has been tested to ensure minimal cross-reaction with bovine, goat, human, mouse, rat, sheep, horse, hamster, guinea pig serum proteins as indicated in https://www.jacksonimmuno.com/catalog/products/711-165-152.
12) DyLight 488-conjugated Donkey Anti Rabbit IgG (Jackson ImmunoResearch,711-486-152) has been tested to ensure minimal cross-reaction with bovine, goat, human, mouse, rat, sheep, horse, hamster, guinea pig serum proteins as indicated in the product Specifications sheet.
13) Alexan Fluor488-conjugated Goat Anti-mouse IgG Fab2 have been optimized for use as a secondary antibody inimmunofluorescent applications as in https://www.cellsignal.com/products/secondary-antibodies/anti-mouse-igg-h-l-f-ab-2-fragment-alexa-fluor-488-conjugate/4408.
14) Alexan Fluor594-conjugated Goat Anti-mouse IgG Fab2 have been adsorbed against human IgG and human serum. Cross-reactivity with rat primary antibodies has been observed as indicated in https://www.cellsignal.com/products/secondary-antibodies/anti-mouse-igg-h-l-f-ab-2-fragment-alexa-fluor-594-conjugate/8890.
15) Anti-rabbit IgG, HRP-linked Antibody#7074S is thoroughly validated with CST primary antibodies and will work optimally with the CST western immunoblotting protocol, ensuring accurate and reproducible results as indicated in https://www.cellsignal.com/product/productDetail.jsp?productId=7074&country=CA.

Eukaryotic cell lines

Policy information about cell lines and Sex and Gender in Research

Cell line source(s)	HEK-293 cells (293T) are from Dr. St-Arnaud's lab at Shriners Hospitial for Children, Canada which were purchased from ATCC CRL-1573.<br>ATDC5 cells are from Dr. Moffatt's lab at Shriners Hospital for Children, Canada which were purchased from Sigmal-Aldrich #99072806.
Authentication	Cell lines were not authenticated.
Mycoplasma contamination	We have not checked for mycoplasma contamination during the last five years. For each of the experiments, same batch of cells were used for both control and experimental groups which were examined in parallel.
Commonly misidentified lines<br>(See ICLAC register)	As indicated in the ICLAC register, HEK cells and a derivative line were reported to be contaminated by HeLa cells, however whether this is applicable to the batch used by us is unknown. ATDC5 cells were not found in the register.

Animals and other research organisms

Policy information about studies involving animals; ARRIVE guidelines recommended for reporting animal research, and Sex and Gender in Research

Laboratory animals	Mice (Mus musculus), 3 weeks or 6 weeks old, male and female were kept in a temperature- (21-22 degree C) and humidity (45%)-controlled room at 12 hr day/night cycle. Food and water were given ad libitum. All procedures were performed using an animal user protocol approved by the Animal Care Committee of McGill University.
Wild animals	No wild animals were used in the study
Reporting on sex	Initial experiments to characterize the skeletal pathologies were performed on both sexes. As we observed no sex-specific traits, male mice were used for subsequent studies.
Field-collected samples	No field-collected samples were used in the study
Ethics oversight	All mouse experiment procedures were approved by the Animal Care Committee of McGill University (Animal Use Protocol 2012-7132) and conducted in accordance with the IACUC's guidelines.

Note that full information on the approval of the study protocol must also be provided in the manuscript.
